# Clinical Review

**Published:** 1839

**Authors:** 


### Clinical Review.

### REPORTS OF LUNATIC ASYLA.

I. Report of the Montrose Lunatic Asylum, Infirmary and Dispensary, for the Year ending 1st of June 1839.

This Report is drawn up by the medical superintendent, Dr. Poole, and is very satisfactorily executed. We do not deem it requisite to go into the Report systematically, much of it referring to points we have often dwelt on. The following observations merit attention, the more, too, as patients are frequently reduced excessively and kept reduced, when sedatives, nay even support, are needed.

"Several of the cases, indeed, having been actively treated, before arrival, with a view to allaying excitement, needed a process totally unlike what is imagined to be the routine of lunatic asylums. They were in hazard of sinking from mere debility, though perhaps as noisy and turbulent as ever. Rest, the warm-bath, generous diet, moderate cordials—possibly opiates and anodynes, &c., according to circumstances—together with free access to the open air, the interdiction of all ascertained annoyances, obvious confidence and kindliness on the part of attendants, were had recourse to, and never so unsuccessfully as to justify another system. It is a serious mistake to believe that the most alarming of the symptoms now enumerated occurred principally in robust subjects, who could bear depletion and a lowering treatment to an indefinite amount. A *Septuagenarian*—pale, withered, feeble, and exhausted—was one of them. I mention him as an example every way *in extremis*, but, though not equalled, it was approximated or had analogies in other instances. He rallied. A much younger man, nearly as debilitated, is now progressing in the same path; but, curiously enough, though clearly thriving, and almost as peaceable as he had been the reverse, he happened to be a humble admirer of the Sangrado doctrine, and insisted on bleeding, which I was sure would have been the death of him. I have no doubt that he will be a convert ere long, for he acknowledges some improvement."

Dr. Poole dwells of course on the advantages of occupation, and practises gentleness and moderate licence. And he reprobates the disinclination of friends to send patients to asyla, till time has gone far to rivet their malady.

II. Nineteenth Annual Report of the Directors of the Dundee Royal Asylum for Lunatics, for the Year ending 31st May, 1839.

In this, as in other instances, we shall merely pick out what particulars are generally interesting.

1. *Occupations of the Lunatics.*—A Table is presented, which exhibits in a strong light the great modern improvement in the treatment of the lunatic—giving him corporeal occupation and mental recreation. The Table we omit because the naked fact is of more consequence than its numerical value; but the following observations bearing on the fact deserve insertion.

"During the erection of the buildings, the patients are busily employed in throwing the foundations, making drains, and in removing earth and rubbish. By their labour several mounds have been erected within the airing grounds, paved on the top, which command a view of the surrounding country, and dispel the monotony and tediousness of a life much secluded from the world.

The occupations of the higher classes are more varied, and their tastes are gratified in every reasonable wish and healthful exercise. Their amusements and recreations have been particularly specified in former Reports: It may be proper, however, now to add, that, for some time, they have been indulged with more frequent excursions into the country; and, for this purpose, open and close carriages are provided for them twice a week. There is one fact that cannot be passed over in silence, as it has excited feelings of surprise in the breasts of those who have most experience in the treatment of the insane: A lady of the highest class some time ago expressed a desire that some children were put under her care,—her wish was gratified. A few children from the neighbourhood attend her daily, and she superintends their education, not only with maternal tenderness but with prudence, temper, and judgment; and, amid the many privations to which she is subject, she is conscious that she is not living in vain, but is employing the means within her power of conferring some benefit on our race.

The Directors have now full experience of the beneficial results that have flowed from the introduction of labour into the Asylum, and they have the satisfaction to know that the practice adopted here is closely followed in a number of similar institutions. There is no doubt that these exercises have increased the number of cures, ameliorated the condition of the patient, and shortened the period of the residence in the House; for there are not a few who are now restored to the peaceful bosom of their own families, happy in the consciousness of conferring benefits on others, who without these means would have been still lingering in the airing grounds, sinking under the heavy burden of imaginary evil, or the pitiable victims of illusory hopes."

2. *Attendance on Religious Worship.*—Aware, says the reporter, that some distinguished writers still entertain doubts of the propriety of introducing public worship into an Asylum, and recommend great caution and prudence in respect to a subject so sacred and exciting as religion, the directors are quite ready to admit that, in regard to a number of the patients, their joining in social religious exercises would be quite discordant to the state of their own minds, and no small disturbance to the pious feelings of others; and though in a few cases it may be difficult to decide as to the propriety of this step, yet the experience of nine years in this Asylum sufficiently proves that, in regard to patients in general, these doubts are imaginary and groundless; and it is believed that, as far as the experiment has been judiciously tried, the same testimony has been uniformly given by the directors of similar institutions.

The average number who have attended the services of the chaplain during the year is one hundred and twenty-five, while a number attend different churches in Dundee for reasons that would be tedious to enumerate.

3. *Impossibility of totally dispensing with Personal Restraint.*—In the recent publication of Mr. Hill of the Lincoln Asylum, he asserts, "that, in a properly-constructed building, with a sufficient number of suitable attendants, restraint is never necessary, never justifiable, and always injurious in all cases of lunacy whatever." It is believed that, if this point were to be settled by practical men, Mr. Hill would be left in a very small minority. But, even allowing the practicability of the measure, whether it be humane and desirable, is a question that appears to be very problematical. If the mind of a patient can be subdued by the power of intimidation, so as to paralyze his efforts in the hour of maniacal paroxysms, would his condition be more happy, or his welfare more promoted, by being under the influence of terror? Is it better to enslave the mind than enchain the body? May there not be greater benevolence and sympathy in subjecting the members of the body to salutary restraint than in the exercise of a moral discipline which will ever appear to human feeling burdensome and oppressive? Instances are not unfrequent of patients, when warned by symptoms of approaching paroxysms, requesting to be placed under temporary restraint; and, when the hour of illusion has passed away, are happy in the reflection that they have been without the power of committing an act which reason and conscience condemn.

With the exception of instances of very violent excitement, where there is strong apprehension of danger to the patient himself, or to others, there has been no restraint ever imposed on the inmates of this House; and, while all necessary precautions are used for the safety of the individual, all compulsatory measures are carefully and anxiously avoided. It sometimes happens that all the patients of every class and in every stage of the disease are free from every kind of restraint whatever; but, as some restraint might occasionally be found necessary among the same number of persons of sound mind, placed in similar circumstances, it is not thought proper that in an establishment of lunatics the life of an individual be placed in jeopardy, or the general comfort of the inmates be disturbed, in support of a theory which seems to aim at ideal perfection rather than to promote practical utility. At the present moment there are only three males and two females subjected to temporary restraint; some of them have no consciousness that they are suffering any evil; and in every society it is unavoidable that a person who disturbs the peace of others suffer for the sake of the public weal.

4. *Two Descriptions of Suicides.*—Among the admissions there was, as usual, a number of suicidal patients; and the same thing has been observed here as in other institutions, that the majority of these cases occur among the females. In some of them, previous to admission, the attempts at self-destruction had nearly been successful; and in too many the attempt seems to have been the first circumstance to awaken the minds of the relatives to the necessity of placing the sufferers under proper restraint. One of them has already been confined no less than six times during the course of the last eighteen years. Like many other patients, with this propensity, when the disease is just commencing, she feels an almost uncontrollable impulse to commit self-destruction, with an almost equal dread of it. For a short period at the beginning of the disease, she is able to withstand the impulse, and her great desire is then to be sent to the Asylum as a place of refuge, where she is certain of being restrained from committing an act so fearful. More than once, however, not having been sent soon enough, the impulse overpowered her, and her attempts were nearly successful. In the majority of these cases we have ascertained that there was a hereditary tendency to insanity.

There is another class of patients who are occasionally suicidal, and who are generally the most difficult patients to manage,—we allude to such as seem from their birth to have a deficiency of moral feeling, with a headlong propensity to various descriptions of vice, frequently combined with a dulness of intellect, which may be termed partial idiocy. Sometimes also there are instances of the same propensity to immortality among those who are well educated and whose intellectual faculties had formerly been perfectly unclouded, but the natural consequence of indulgence in habits of immortality is to reduce them also after a time to a state of partial, sometimes of total idiocy. Our experience tells us that patients of this class are seldom to be depended upon. They seem to have some obliquity which does not permit them to distinguish between right and wrong, and either lack memory or are incapable of performing their promises, by being as it were impelled to commit acts of folly and intemperance. We are persuaded that this variety of insanity is often called into action by want of proper education and proper training in early youth, and that, in many cases, by judicious conduct and salutary discipline, derangement might have been prevented. It would have been well for many such patients that, instead of having a competency, they had been obliged to earn a livelihood by the sweat of their brow.

5. *Taciturnity cured by a Blister.*—"The patient was a man of high moral character. Before he was sent here he had an attack of general mania, succeeded by weakness, torpidity, and taciturnity. In the latter state we admitted him in the Autumn of 1837. For several months afterwards, on being repeatedly urged, he would say, 'Yes, or no,' but without a proper knowledge of his reply; but for a long time past we could not get even a monosyllable from him. His appetite fell off for some time: he became weak and emaciated, and would take scarcely any nourishment, but a little wine and beef-tea daily. At last he was obliged to keep his bed; and, to rouse him, we tried a blister on the neck. The day following its application, he looked more lively, and attempted to speak to his attendant: His wife was immediately sent for; and, when she came, the spell that bound his tongue entirely gave way, and he talked as collectedly and sanely as ever he did in his life. On being asked why he had not spoken for such a length of time, he replied that he had not had the power to do so, for that something always prevented him, and that it was the blister that restored him to speech and consciousness. He has been discharged well."

6. As a *Cause* hereditary predisposition is still found in the foremost rank.

7. *Mild Remedies very necessary.*—" It is a mistake to suppose that lunatics indiscriminately require larger doses of medicine than others. There are patients here who would be materially injured by more than a tea-spoonful of castor oil or Gregory's powder, and powerful men whose stomachs cannot bear antimony even in the smallest dose. One patient experienced fits, by night as well as by day, of distressing and loud eructation, which was speedily relieved by gentle medicine and the shower-bath, and then an effectual cure rapidly followed. For certain patients in some Asylums we see a bath made use of called the "Bath of Surprise." It is unknown here, but from its description we think it a very questionable remedy. Local blood-letting in small quantities, repeated according to circumstances, dry cupping, and in one case the application of a seton to the nape of the neck, have been of the most essential service. One gentleman whose malady was caused by debauchery, and who is subject to violent paroxysms, and takes every opportunity to attempt to commit a *private vice*, has been much benefitted by having his occiput regularly shaved and frequently sponged with cold water."

### III. MEDICAL REPORT TO THE MANAGERS OF THE LUNATIC ASYLUM OF ABERDEEN, FOR THE YEAR ENDING 30TH APRIL, 1839.

The Reporters, Drs. Macrobin and M'Kinnon, observe that—the number of cases admitted into the Aberdeen Asylum during the last year, amounting to 53, has been larger than during any former year. This increase may be accidental; but it is more probable that insanity is not only more commonly met with in society, from the growing nature of the population, but that it is so relatively, from the operation of that law peculiar to it, with a few other diseases, *viz.*, its hereditary transmission from one generation to another. And the law just alluded to is, perhaps, stronger, in regard to insanity, than almost any other malady. As of late years much improvement has been effected (both at home and abroad) in regard to the treatment of a disease which used, at one time, to be considered beyond the reach of medical science,—so there is reason to hope this improvement may continue to keep pace with its growing frequency. And thus, in the event of insanity becoming more common, it will also be more frequently cured. Considering also the gradual change taking place in the public mind, in regard to the subject of insanity in general, we confidently trust that, ere long, that foolish prejudice will be got rid of, which, by leading, in many instances, the friends, as well as the patients, to conceal the malady as long as possible even from the physician, throws one of the greatest barriers in the way of the recovery of the insane; the early and curable stage being allowed to pass away without any attempt at rational treatment, or, at best, under the trial of ill-directed and inefficient measures.

The greater number of cases occurred between the ages of 20 and 40, that period when excitements of all kinds are most rife.

Of the 53 cases the proportions were—

| | Males. | Females. | Total. |
|---|---|---|---|
| Mania .. .. .. .. | 5 | 8 | 13 |
| Monomania, *with excitement* .. | 7 | 7 | 14 |
| Monomania, *with depression* .. | 5 | 8 | 13 |
| Dementia and Fatuity .. .. | 7 | 5 | 12 |
| Moral Insanity .. .. .. | 1 | 0 | 1 |
| | 25 | 28 | 53 |

The Reporters remark that the monomaniacs were seldom purely *monomaniac*, the judgment being usually perverted upon other points.

They remark that, in the last report, allusion was made to two cases of *moral insanity*, or that form of the disease which is accompanied by perversion of the *moral*, without corresponding derangement of the *intellectual*, faculties. Another case has since then come under our notice, in which the means found useful in other cases of insanity effected a recovery, viz.—removal from the scenes and persons that tended to keep up excited feelings, together with the exercise of moral discipline, and appropriate medical treatment. Such cases are interesting in certain circumstances, from their medico-legal bearing on responsibility.

*Causes.*—A Table is given for the purpose of conveying some idea of these. Hereditary predisposition was traced in ten cases. Intemperance, observe the Reporters, in the use of ardent spirits was found to be the exciting cause in five of the cases. In the first stage of insanity, cordials are frequently had recourse to, in the vain hope of removing unwonted despondency, while they but too often precipitate the attack. Overstrained mental application among men, especially when accompanied by *anxiety*, and overstrained and perverted devotion in females, are not unfrequent causes of insanity. But, amongst the latter class, perhaps the most frequent of all causes are uterine irritations and derangements. Fortunately, the primary disorder is often amenable to medical treatment, in which case the secondary affection of the brain usually subsides; unless, from neglect, that which was functional has assumed the character of organic disease. In a few instances, the insanity was an effect of a previously diseased state of the nervous system, which had manifested itself in the way of apoplexy or palsy. The mental disorder in these cases, almost invariably put on the form of dementia, or fatuity, from the first; and the patients generally betrayed a propensity to violence.

### *Mortality and Morbid Anatomy.*

"Fourteen patients died during the last year. Five died of *general palsy;* and it may be noticed that these were all male patients;—thus confirming what has been remarked so often in former Reports, and what corresponds also with the experience of other institutions, viz., that such palsy is relatively more common in men. Three died of consumption—a disease frequently met with among the inmates of an Asylum. Two females, of weak constitution, sank under the exhaustion consequent on a violent and prolonged stage of excitement. Two died apoplectic. One died of dropsy; and one of inflammation of the lungs. In all, with one exception, (where the consent of the friends could not be obtained) an examination of the body was made after death. The morbid appearances corresponded in most respects with those noticed in former Reports, and tended to confirm us in the opinion that insanity is not necessarily or essentially of an inflammatory nature in its early stage, though it rarely fails to lead to inflammation, and its products, when it has continued long. Sometimes the most violent maniacal excitement appears in connexion with a condition of the nervous and vascular systems, in which the patient is calmed, rather than excited, by the cautious exhibition of stimulants; and which leaves, after death, no morbid appearances corresponding to the violence of the symptoms during life."

These latter observations we deem important. The excitement of mania is too often looked on as the excitement produced by vascular action, and treated mischievously in consequence of that view. The morbid appearances after death are thus stated.

Opacity of the arachnoid, and a copious effusion of serum, sometimes milky—presenting the aspect of a gelatinous layer over the surface of the brain—were the most common of the morbid appearances; and never failed to be well marked in those who, during life, had exhibited paralytic symptoms. As regards the other organs of the body, disease of the heart, generally in the form of hypertrophy of the left ventricle, was frequently found. Such a condition of the organ must, no doubt, in some instances, have operated as a remote cause of insanity, by exciting, and otherwise interfering with the regular distribution of the blood within the head; while, in other cases, the strong emotions of the mind, and violent muscular exertions—the result of an excited state of the brain—may have re-acted on the heart, and the disease of the latter been in this way an effect, rather than a cause of the insanity. In one of the patients, whose delusions, during life, were of a hypochondriacal nature, the morbid appearances were instructive; for, while the traces of morbid action within the head were slight, an unusual arrangement of the viscera of the abdomen was found, the transverse arch of the colon being tied down to the rectum by a morbid adhesion, so as at all times to have interfered with the functions of the intestinal canal. The symptoms, during life, also indicated some abdominal lesion.

Of occupation the Reporters of course speak favourably. It is, when judiciously prescribed and pursued, a very admirable remedy.

We can only add, in reference to all these reports, that we are pleased at their appearance. How much the treatment of insanity has improved. How reasonable it is to suppose that it will improve still more. The Reporters deserve thanks for their zeal.

